# Accessibility of Telehealth Services During the COVID-19 Pandemic: A Cross-Sectional Survey of Medicare Beneficiaries

**DOI:** 10.5888/pcd18.210056

**Published:** 2021-07-01

**Authors:** Boon Peng Ng, Chanhyun Park

**Affiliations:** 1 College of Nursing, University of Central Florida, Orlando, Florida; 2 Disability, Aging, and Technology Cluster, University of Central Florida, Orlando, Florida; 3 Department of Pharmacy and Health Systems Sciences, School of Pharmacy, Northeastern University, Boston, Massachusetts; 4 Now affiliated with Health Outcomes Division, College of Pharmacy, University of Texas at Austin, Austin, Texas

## Abstract

**Introduction:**

Telehealth plays a role in the continuum of care, especially for older adults during the COVID-19 pandemic. Our objective was to examine factors associated with the accessibility of telehealth services during the COVID-19 pandemic among older adults.

**Methods:**

We analyzed the nationally representative Medicare Current Beneficiary Survey COVID-19 Rapid Response Supplement Questionnaire of beneficiaries aged 65 years or older. Two weighted multivariable logistic regression models were used to examine associations between usual providers who offered telehealth 1) during the COVID-19 pandemic and 2) to replace a regularly scheduled appointment. We examined factors including sociodemographic characteristics, comorbidities, and digital access and literacy.

**Results:**

Of the beneficiaries (n = 6,172, weighted n = 32.4 million), 81.2% reported that their usual providers offered telehealth during the COVID-19 pandemic. Among those offered telehealth services, 56.8% reported that their usual providers offered telehealth to replace a regularly scheduled appointment. Disparities in accessibility of telehealth services by sex, residing area (metropolitan vs nonmetropolitan), income level, and US Census region were observed. Beneficiaries who reported having internet access (vs no access) (OR, 1.75, *P* < .001) and who reported ever having participated in video, voice, or conference calls over the internet before (vs not) (OR, 2.18, *P* < .001) were more likely to report having access to telehealth. Non-Hispanic Black beneficiaries (versus White) (OR, 1.57, *P* = .007) and beneficiaries with comorbidities (vs none) (eg, 2 or 3 comorbidities, OR, 1.25, 95% *P* = .044) were more likely to have their usual provider offer telehealth to replace a regularly scheduled appointment.

**Conclusion:**

Although accessibility of telehealth has increased, inequities raise concern. Educational outreach and training, such as installing and launching an online web conferencing platform, should be considered for improving accessibility of telehealth to vulnerable populations beyond the COVID-19 pandemic.

**Summary**

**What is already known on this topic?**

Factors associated with accessibility and use of telehealth have been reported for the adult population; however, little is known about factors associated with accessibility of telehealth among older adults.

**What is added by this report?**

Over 80% of Medicare beneficiaries in our study reported that their usual providers offered telehealth during the COVID-19 pandemic. Disparities in accessibility of telehealth services by sex, residing area, income, and census region were observed.

**What are the implications for public health practice?**

Educational outreach and training, such as improving digital literacy, can be considered for improving accessibility of telehealth during the COVID-19 pandemic and beyond.

## Introduction

Since the first documented community spread of COVID-19 in the US on February 26, 2020, the pandemic has affected many (1). As of February 9, 2021, the total number of COVID-19 cases in the US was approximately 27 million, with the number of deaths exceeding 460,000 (2). Within the Medicare population, as of November 2, 2020, about 1.9 million total COVID-19 cases and over 493,000 COVID-19–related hospitalizations had been reported (3). Many studies reported higher adverse health outcomes, such as mortality and hospitalization among older adults with COVID-19 (4–6). A study reported that among recorded deaths, about 80% were adults aged 65 years or older, often with chronic conditions (5). Furthermore, COVID-19 imposes a substantial economic burden. As of November 2, 2020, Medicare spent $7.4 billion in fee-for-service claims alone for COVID-19–related hospitalizations, with an average of $23,558 per beneficiary (3). Research related to older adults with COVID-19, therefore, remains a high priority.

With the rapid spread of COVID-19 that has affected everyday life, preventive behaviors, such as social distancing, mask wearing, and handwashing, have been recommended by health care organizations (7,8). At various phases of the pandemic, many US states mandated or encouraged their residents to minimize the risk and spread of COVID-19 (9). Sheltering-in-place, however, presents a dilemma for vulnerable populations, such as older adults with chronic conditions that require a regular continuum of care, because they must choose between risking COVID-19 exposure and delaying care. As a result, the Centers for Disease Control and Prevention has recommended that providers offer care via telehealth (1,10). Telehealth is the use of 2-way telecommunication technologies to provide clinical health care through a variety of remote methods (1). Telehealth came to recognition as a vital mode of care delivery during the pandemic, especially for older adults at high risk of adverse health outcomes from COVID-19 (11). During the early months of the pandemic, studies on the availability and use of telehealth showed a rapid increase of telehealth use (1,12,13). These studies reported that younger patients and female patients had the most telehealth encounters (1), and race and income disparities in the use of telehealth at the zip code level were observed (13).

Although the availability and use of telehealth is longstanding, there have been barriers to widespread use, such as lack of infrastructure, strict regulation, and sparse reimbursement structure (14,15). With the passing of the Coronavirus Preparedness and Response Supplemental Appropriations Act, the US Department of Health and Human Services via the Centers for Medicare and Medicaid Services (CMS) was able to authorize policy changes and regulatory waivers in March 2020. These interventions focused on Medicare-related requirements for telehealth services, thereby applying no penalties for using technologies not compliant with the Health Insurance Portability and Accountability Act, and waivers were provided for use of telehealth services for Part B beneficiaries to broaden and facilitate the use of telehealth (15). In response to these changes, information about access to telehealth among Medicare beneficiaries is of wide interest. The objectives for our study, therefore, were 1) to examine factors (ie, sociodemographic; comorbidity; access to technology and the internet; and previous experience with video, voice, or conference calls over the internet) associated with having health care providers who offered telehealth to regular patients during the COVID-19 pandemic, and 2) to examine factors associated with providers who offered telehealth to replace a regularly scheduled appointment for Medicare beneficiaries aged 65 years or older.

## Methods

We used data from the 2020 Medicare Current Beneficiary Survey (MCBS) COVID-19 Summer Supplemental Public Use File for our study (16). The MCBS COVID-19 Summer Supplement was administered from June 10, 2020, through July 15, 2020, to existing MCBS beneficiaries by telephone, with an overall response rate of 78.9% (16). The supplement surveyed Medicare beneficiaries (either themselves or through a proxy respondent) who were continuously enrolled in Medicare from January 1, 2020, and who were alive and living in the community in the summer of 2020 (16). Survey data are nationally representative and cross-sectional of community-dwelling Medicare beneficiaries. The MCBS COVID-19 Summer Supplement collected data on the effects of the COVID-19 pandemic on Medicare beneficiaries, such as availability and use of telehealth, access to technology devices and the internet, and other COVID-19–related variables. The survey was conducted in either English or Spanish. Additional information related to the survey is available and published at the CMS MCBS website (16).

Our study population included community-dwelling Medicare beneficiaries aged 65 years or older who responded to questions regarding their usual providers who offered telehealth during the COVID-19 pandemic (n = 6,172 and weighted n = 32.4 million), and regarding their usual providers who offered telehealth to replace a regularly scheduled appointment (n = 4,692 and weighted n = 25.6 million).

### Measures

The accessibility of telehealth was measured by the following questions (16):

1. Does [your/(SP [sample person])’s] usual provider offer telephone or video appointments, so that [you don’t/he/she doesn’t] need to physically visit their office or facility?
2. Did [your/(SP)’s] usual provider offer [you/him/her] a telephone or video appointment to replace a regularly scheduled appointment **during the coronavirus outbreak**?

We created a binary variable of access to telehealth during the COVID-19 pandemic with the value of 1 for those who responded yes and 0 for those who responded No to question 1. Only those who responded yes to question 1 were then asked question 2 (those who responded yes were coded as 1; those who responded no were coded as 0). The usual provider is a particular doctor or other health professional beneficiaries usually go to when they are sick or ask for advice about their health.

The independent variables included were guided by previous studies (1,12,13,17) and the Andersen Behavioral Model of Health Services Use (18), which hypothesizes that predisposing, enabling, and need-related factors affect the use of health care services. For predisposing factors, we included age (65–74 y, ≥75 y), sex (male, female), race/ethnicity (non-Hispanic White, non-Hispanic Black, Hispanic, and other [Asian, Native Hawaiian or Pacific Islander, Indian or Alaska Native, multi-racial, unknown]), and language other than English spoken at home (yes, no). For enabling factors, we included residing area (metropolitan, nonmetropolitan); living status (alone, not alone); US Census region (Northeast, Midwest, South, West); annual income level (<$25,000, ≥$25,000); access to the internet (yes, no); own or use either a desktop or laptop computer, a smartphone, or a tablet (yes, no) (this binary variable was recoded and created based on an individual variable of owning or using each of those 3 types of devices); and whether the beneficiary had previously participated in video, voice, or conference calls over the internet (yes, no). For need-related factors, we included a categorical variable for the number of comorbidities (≤1, 2 or 3, 4 or 5, or ≥6). For the number of comorbidities, the following health conditions were included: high blood pressure; high cholesterol; myocardial infarction; angina; congestive heart failure; other heart conditions; arthritis; diabetes; depression; emphysema, asthma, or chronic obstructive pulmonary disease; Alzheimer or dementia; osteoporosis; cancer (nonskin); and stroke or brain hemorrhage.

### Statistical analyses

We used a cross-tabulation analysis with Wald χ^2^ tests to examine the differences in the proportions of beneficiaries who reported that their usual providers offered telehealth during the COVID-19 pandemic and offered telehealth to replace a regularly scheduled appointment, by sociodemographics, comorbidities, and access to technology devices and the internet. Two multivariable logistic regression models were used to examine the association between usual providers who offered telehealth 1) during the COVID-19 pandemic and 2) to replace a regularly scheduled appointment. Factors examined were sociodemographic characteristics, comorbidities, access to technology devices and the internet, and having previously participated in video, voice, or conference calls over the internet. All analyses used the survey weights from the data set to account for the complex survey design (16). SAS Enterprise Guide version 6.1 (SAS Institute, Inc) and Stata/MP version 16.1 (StataCorp LLC) were used to perform the analysis.

## Results

Of study beneficiaries aged 65 years or older, 81.2% (representing approximately 26.3 million beneficiaries) reported that their usual providers offered telehealth during the COVID-19 pandemic (Table 1). A higher proportion of younger beneficiaries (aged 65–74 y) than older beneficiaries (84.2% vs 76.1%, *P* < .001) reported that their usual providers offered telehealth during the pandemic (Table 1). Beneficiaries living in a metropolitan area (vs nonmetropolitan area, 82.9% vs 73.1%, *P* < .001) and census regions other than South (eg, for the West 85.5% vs 77.2% for the South, *P* = .007) reported higher prevalence of their usual providers offering telehealth during the pandemic. More beneficiaries with higher income (≥ $25,000) reported that their usual providers offered telehealth during the pandemic compared with lower income beneficiaries (<$25,000) (85.0% vs 69.8%, *P* < .001). More usual providers offered telehealth during the pandemic for beneficiaries who had access to internet (vs those without access) (84.7% vs 62.1%, *P* < .001), who had access to technology devices (vs those with no access) (84.0% vs 65.6%, *P* < .001), and who had previously participated in video, voice, or conference calls over the internet (vs those who did not) (89.0% vs 71.3%, *P* < .001) (Table 1).

**Table 1:** Characteristics of Medicare Beneficiaries Aged 65 Years or Older With Usual Providers Who Offered Telehealth During the COVID-19 Pandemic and Offered Telehealth to Replace a Regularly Scheduled Appointment, Medicare Current Beneficiary Survey, COVID-19 Summer Supplemental File, 2020

Variable	Providers Offered Telehealth During the COVID-19 Pandemic				Providers Offered Telehealth to Replace a Regularly Scheduled Appointment^a^			
	Total Weighted % (n; Weighted n)	n (Weighted n)	Weighted %	*P* Value	Total Weighted % (n, Weighted n)	n (Weighted n)	Weighted %	*P* Value
**Total**	100.0 (6,172; 32.4 million)	4,837 (26.3 million)	81.2		100.0 (4,692; 25.6 million)	2,685 (14.5 million)	56.8	
**Age group, y**								
65–74	63.0 (2,694; 20.4 million)	2,235 (17.2 million)	84.2	<.001	65.6 (2,182; 16.8 million)	1,236 (9.5 million)	56.4	.47
≥75	37.0 (3,478; 12.0 million)	2,602 (9.1 million)	76.1		34.4 (2,510; 8.8 million)	1,449 (5.0 million)	57.4	
**Sex**								
Female	54.7 (3,419; 17.7 million)	2,699 (14.5 million)	82.0	.12	55.2 (2,615; 14.2 million)	1,508 (8.0 million)	56.7	.93
Male	45.3 (2,753; 14.7 million)	2,138 (11.8 million)	80.2		44.8 (2,077; 11.4 million)	1,177 (6.5 million)	56.8	
**Race/ethnicity**								
Non-Hispanic White	77.3 (4,696; 25.1 million)	3,759 (20.8 million)	83.0	<.001	79.0 (3,636; 20.2 million)	2,021 (11.1 million)	55.1	.005
Non-Hispanic Black	8.7 (500; 2.8 million)	351 (2.1 million)	74.1		7.8 (339; 2.0 million)	221 (1.3 million)	66.0	
Hispanic	7.8 (646; 2.5 million)	482 (1.9 million)	74.4		7.3 (476; 1.8 million)	292 (1.1 million)	61.3	
Other	6.2 (330; 2.0 million)	245 (1.5 million)	76.6		5.9 (241; 1.5 million)	151 (0.9 million)	60.9	
**Residing area**								
Metropolitan	82.0 (4,861; 26.6 million)	3,926 (22.1 million)	82.9	<.001	83.7 (3,808; 21.4 million)	2,188 (12.2 million)	57.0	.52
Nonmetropolitan	18.0 (1,311; 5.8 million)	911 (4.3 million)	73.1		16.3 (884; 4.2 million)	497 (2.3 million)	55.4	
**Living status**								
Alone	19.0 (1,316; 6.2 million)	997 (4.8 million)	78.7	.058	18.5 (973; 4.7 million)	550 (2.7 million)	56.1	.74
Not alone	81.0 (4,856; 26.3 million)	3,840 (21.5 million)	81.7		81.5 (3,719; 20.9 million)	2,135 (11.9 million)	56.9	
**Census region**								
Northeast	18.2 (1,078; 5.9 million)	858 (4.8 million)	82.1	.007	18.4 (831; 4.7 million)	492 (2.8 million)	59.4	.31
Midwest	21.8 (1,377; 7.1 million)	1,086 (5.8 million)	82.2		22.1 (1,057; 5.7 million)	589 (3.1 million)	55.2	
South	36.1 (2,283; 11.7 million)	1,685 (9.0 million)	77.2		34.1 (1,626; 8.7 million)	951 (5.0 million)	57.6	
West	23.9 (1,434; 7.8 million)	1,208 (6.6 million)	85.5		25.4 (1,178; 6.5 million)	653 (3.6 million)	55.0	
**Language other than English spoken at home**								
Yes	11.3 (823; 3.7 million)	614 (2.8 million)	75.4	.012	10.6 (600; 2.7 million)	374 (1.7 million)	61.7	.055
No	88.7 (5,349; 28.7 million)	4,223 (23.5 million)	81.9		89.4 (4,092; 22.9 million)	2,311 (12.8 million)	56.2	
**Annual income level, $**								
<25,000	25.1 (1,895; 8.1 million)	1,304 (5.7 million)	69.8	<.001	21.6 (1,267; 5.5 million)	762 (3.3 million)	60.2	.02
≥25,000	74.9 (4,277; 24.3 million)	3,533 (20.6 million)	85.0		78.4 (3,425; 20.0 million)	1,923 (11.2 million)	55.8	
**Access to internet**								
Yes	84.5 (4,854; 27.4 million)	4,029 (23.2 million)	84.7	<.001	88.3 (3,918; 22.6 million)	2,226 (12.7 million)	56.2	.06
No	15.5 (1,318; 5.0 million)	808 (3.1 million)	62.1		11.7 (774; 3.0 million)	459 (1.8 million)	60.5	
**Previously participated in video, voice, or conference calls over the internet**								
Yes	55.8 (2,984; 18.1 million)	2,623 (16.1 million)	89.0	<.001	61.4 (2,558; 15.7 million)	1,492 (9.0 million)	57.2	.44
No	44.2 (3,188; 14.3 million)	2,214 (10.2 million)	71.3		38.6 (2,134; 9.9 million)	1,193 (5.5 million)	56.0	
**Own or use computer, smart phone, or tablet**								
Yes	84.3 (4,792; 27.3 million)	3,931 (23.0 million)	84.0	<.001	87.5 (3,820; 22.4 million)	2,163 (12.6 million)	56.3	.13
No	15.7 (1,380; 5.1 million)	906 (3.3 million)	65.6		12.5 (872; 3.2 million)	522 (1.9 million)	59.9	
**No. of chronic conditions**								
0 or 1	15.6 (771; 5.1 million)	631 (4.2 million)	84.3	<.001	16.2 (610; 4.1 million)	290 (1.9 million)	47.0	<.001
2 or 3	38.3 (2,266; 12.4 million)	1,796 (10.3 million)	82.6		38.9 (1,734; 10.0 million)	907 (5.2 million)	52.5	
4 or 5	30.8 (2,021; 10.0 million)	1,562 (8.0 million)	79.5		30.4 (1,528; 7.8 million)	920 (4.7 million)	60.8	
≥6	15.3 (1,114; 4.9 million)	848 (3.8 million)	77.5		14.5 (820; 3.7 million)	568 (2.6 million)	70.3	

a Only applied to beneficiaries who responded yes to the question “Does [your/(SP [sample person])’s] usual provider offer telephone or video appointments, so that [you don’t/he/she doesn’t] need to physically visit their office or facility?” during the COVID-19 pandemic. Only beneficiaries who answered yes to question 1 (the first dependent variable) were then asked question 2, “Did [your/(SP)’s] usual provider offer [you/him/her] a telephone or video appointment to replace a regularly scheduled appointment during the coronavirus outbreak?” However, some beneficiaries answered “don’t know” to question 2. Therefore, there was a difference in the number of beneficiaries (N= 4,837) who answered yes to the question 1 and the number beneficiaries included in the analysis for question 2 (the second dependent variable) (N = 4,692).

Among those being offered telehealth services, 56.8% (approximately 14.5 million beneficiaries) reported that their usual providers offered telehealth to replace a regularly scheduled appointment (Table 1). Non-Hispanic Black beneficiaries reported a higher prevalence of having their usual providers offer telehealth to replace a regularly scheduled appointment (66.0%) than those of other races/ethnicities (eg, for non-Hispanic White beneficiaries, 55.1%, *P* = .005) (Table 1). Compared with beneficiaries without comorbidities, those with comorbidities reported a higher prevalence of having their usual providers offer telehealth to replace a regularly scheduled appointment (eg, 47.0% for those with ≤1 chronic condition vs 52.5% for those with 2 or 3 chronic conditions, *P* < .001).

Of study beneficiaries aged 65 or older, those aged 65 to 74 had 1.29 times the odds (95% CI, 1.09–1.53; *P* = .003) of having their usual providers offer telehealth during the COVID-19 pandemic than those aged 75 or older (Table 2). Male beneficiaries had 0.80 times the odds (95% CI, 0.68–0.94; *P* = .006) of being offered telehealth services during the COVID-19 pandemic than female beneficiaries. Beneficiaries living in a metropolitan area were more likely to report being offered telehealth services during the COVID-19 pandemic than those living in a nonmetropolitan area (OR, 1.56; 95% CI, 1.18–2.05; *P* = .002). Beneficiaries from the West and Midwest census regions were more likely to report that their usual providers offered telehealth during the COVID-19 pandemic than those from the South. Beneficiaries who reported an income level below $25,000 were less likely (OR, 0.71; 95% CI, 0.58–0.86; *P* = .001) to report that their usual providers offered telehealth during the COVID-19 pandemic than those whose income was higher. Those who reported having access to the internet and having previously participated in video, voice, or conference calls over the internet had 1.75 (95% CI, 1.41–2.18; *P* < .001) and 2.18 (95% CI, 1.78–2.67; *P* < .001) times the odds of having their usual providers offer telehealth services during the COVID-19 pandemic than their counterparts, respectively.

**Table 2:** Factors Associated With Usual Providers Who Offered Telehealth During the COVID-19 Pandemic and Offered Telehealth to Replace a Regularly Scheduled Appointment Among Medicare Beneficiaries Aged 65 Years or Older, Medicare Current Beneficiary Survey, COVID-19 Summer Supplemental File, 2020

Variable	Providers Offered Telehealth During the COVID-19 Pandemic		Providers Offered Telehealth to Replace a Regularly Scheduled Appointment^a^	
	Odds Ratio (95% CI)	*P* Value	Odds Ratio (95% CI)	*P* Value
**Age group, y**				
65–74	1.29 (1.09–1.53)	.003	1.02 (0.89–1.16)	.74
≥75	1 [Reference]		1 [Reference]	
**Sex**				
Female	1 [Reference]		1 [Reference]	
Male	0.80 (0.68–0.94)	.006	1.03 (0.88–1.20)	.68
**Race/ethnicity**				
Non-Hispanic White	1 [Reference]		1 [Reference]	
Non-Hispanic Black	0.89 (0.67–1.17)	.39	1.57 (1.13–2.17)	.007
Hispanic	0.88 (0.64–1.22)	.46	1.12 (0.79–1.58)	.52
Other	0.78 (0.56–1.08)	.14	1.23 (0.87–1.74)	.24
**Residing area**				
Metropolitan	1.56 (1.18–2.05)	.002	1.00 (0.82–1.22)	.96
Nonmetropolitan	1 [Reference]		1 [Reference]	
**Living status**				
Alone	1.03 (0.84–1.26)	.79	0.96 (0.78–1.18)	.68
Not alone	1 [Reference]		1 [Reference]	
**Census region**				
Northeast	1.25 (0.99–1.58)	.06	1.12 (0.90–1.39)	.30
Midwest	1.32 (1.04–1.69)	.03	0.95 (0.80–1.14)	.64
South	1 [Reference]		1 [Reference]	
West	1.54 (1.11–2.13)	.01	0.92 (0.78–1.08)	.31
**Language other than English spoken at home**				
Yes	0.84 (0.59–1.20)	.34	1.16 (0.84–1.60)	.36
No	1 [Reference]		1 [Reference]	
**Annual income level, $**				
<25,000	0.71 (0.58–0.86)	.001	1.03 (0.86–1.22)	.75
≥25,000	1 [Reference]		1 [Reference]	
**Access to internet**				
Yes	1.75 (1.41–2.18)	<.001	0.89 (0.67–1.18)	.42
No	1 [Reference]		1 [Reference]	
**Previously participated in video, voice, or conference calls over the internet**				
Yes	2.18 (1.78–2.67)	<.001	1.21 (1.05–1.39)	.009
No	1 [Reference]		1 [Reference]	
**Own or use computer, smart phone, or tablet**				
Yes	0.94 (0.73–1.22)	.66	1.00 (0.74–1.35)	.98
No	1 [Reference]		1 [Reference]	
**Number of chronic conditions**				
0 or 1	1 [Reference]		1 [Reference]	
2 or 3	0.94 (0.72–1.22)	.62	1.25 (1.01–1.57)	.04
4 or 5	0.83 (0.64–1.09)	.19	1.77 (1.34–2.35)	<.001
≥6	0.86 (0.64–1.16)	.34	2.71 (2.07–3.54)	<.001

a Only applied to beneficiaries who responded yes to the question about providers offering telehealth during the COVID-19 pandemic.

Compared with non-Hispanic White beneficiaries, non-Hispanic Black beneficiaries had 1.57 times the odds of having their usual providers offer telehealth to replace a regularly scheduled appointment (95% CI, 1.13–2.17; *P* = .007) (Table 2). Beneficiaries who had participated in video, voice, or conference calls over the internet before had 1.21 times the odds of having their usual providers offered telehealth to replace a regularly scheduled appointment (95% CI, 1.05–1.39; *P* = .009), compared with those who had not. Having more comorbidities was associated with a higher likelihood of having usual providers who offered telehealth to replace a regularly scheduled appointment (eg, those with 2 or 3 chronic conditions had 1.25 times the odds [95% CI, 1.01–1.57; *P* = .04] of being offered telehealth to replace a regularly scheduled appointment than those with 1 or no chronic condition) (Table 2).

## Discussion

The rapid spread of COVID-19, along with CMS waivers and recent policy changes on telehealth, has accelerated the adoption of telehealth services to prevent and reduce the risk of exposure (15,19). Studies have reported a surge of telehealth availability and use as a result (1,12,13). We found a similar pattern in terms of accessibility of telehealth for Medicare beneficiaries, with approximately 81% of study Medicare beneficiaries aged 65 or older reporting that their usual providers offered telehealth during the COVID-19 pandemic. Among those having access to telehealth, 56% reported that their usual providers offered telehealth to replace a regularly scheduled appointment. Additionally, disparities (ie, sex, residing area, income level, and census region) in accessibility of telehealth services were observed in our study. Beneficiaries who had access to the internet and had previously participated in video, voice, or conference calls over the internet were more likely to report accessibility of telehealth during the COVID-19 pandemic. Non-Hispanic Black beneficiaries and beneficiaries with comorbidities were more likely to report that their usual providers offered telehealth to replace a regularly scheduled appointment than were non-Hispanic White beneficiaries or beneficiaries with no comorbidities. These findings can inform decision makers on the outreach efforts needed for at-risk older populations to improve accessibility of telehealth, perhaps even beyond the pandemic.

Results from our study highlight that factors such as the sex of the beneficiary play an important role in the reported accessibility of telehealth among Medicare beneficiaries. We found that women were more likely to report that their usual providers offered telehealth during the COVID-19 pandemic than men were. This accessibility pattern of telehealth is consistent with a recent report (1) and is likely related to risk aversion, which has been found to be associated with women. Other reports show that women are more concerned about the risk of contracting COVID-19 and are more likely to follow guidelines and preventive measures related to COVID-19 than men are (20,21). Therefore, female beneficiaries were probably more likely to inquire about the use of and to take advantage of telehealth during the COVID-19 pandemic to prevent and reduce the risk of exposure. These findings highlight the need to continue advocating for tailored interventions by sex to improve access to telehealth services for the targeted populations.

Our study also showed that Medicare beneficiaries with low incomes and those living in a nonmetropolitan area or the South were less likely to report accessibility of telehealth during the COVID-19 pandemic than their counterparts. This finding is consistent with a previous study by Jaffe and colleagues (12) that analyzed health inequalities in the use of telehealth in the lens of COVID-19. The authors found that, among adults aged 18 years or older, those living in an urban area and those living in the Northeast, Midwest and West census regions were more likely to have telehealth encounters than those living in rural areas and the South (12). A potential reason could be that some southern states have implemented later and less restrictive stay-at-home orders (9,12,22). Therefore, implementing use of telehealth for Medicare beneficiaries in the South might not seem to be as urgent as in states in other regions. Additionally, lower use of telehealth is associated with areas of economic deprivation (12,23,24). Southern states have the lowest median household income and the highest percentage of people living in poverty in the US (12,25), thereby providing another potential reason for the slower uptake of telehealth in this region. The lack of availability and use of telehealth services in the South, as well as other areas of economic deprivation (ie, rural communities), is particularly concerning considering the rise of COVID-19 cases in these areas. Lewis and colleagues found that those in areas of Utah with high social and economic inequities were more likely to have a COVID-19 infection and be subsequently hospitalized (26). Therefore, sociodemographic disparities in accessibility of telehealth services among Medicare beneficiaries should raise concern among decision makers, particularly because telehealth, at its basis, is designed to overcome access barriers and to reach patients more efficiently and cost effectively (11).

Amid reports that racial and ethnic minority populations have more adverse health outcomes and are disproportionately affected by COVID-19 (3,5), we found that non-Hispanic Black beneficiaries were more likely to report that their usual providers offered telehealth to replace a regularly scheduled appointment than non-Hispanic White beneficiaries. This finding probably reflects the advocacy from many about the effects of COVID-19 on racial and ethnic minority populations to ensure adequate public health resources are allocated and focused on this population. Awareness of the issue, willingness of both providers and patients to use telehealth (27), and efforts from CMS and regulatory bodies such as policy changes on telehealth (ie, 1135 waivers) (15), are important to ensure the availability and use of telehealth for Medicare beneficiaries.

Our study found that Medicare beneficiaries with comorbidities were more likely to report that their usual providers offered telehealth to replace a regularly scheduled appointment, but those with comorbidities also reported lower, although nonsignificant, odds of their usual providers offering telehealth during the COVID-19 pandemic. Patients with multiple comorbidities are more likely to be prioritized to been seen in clinic because of a need for closer medical attention and examination compared with less complicated patients for whom telehealth will suffice. However, for patients with high comorbidity and for whom telehealth was offered and presumably deemed appropriate (ie, answered yes to question 1), higher comorbidity was associated with greater accessibility to telehealth to replace a regularly scheduled appointment (question 2), probably to prevent exposure to COVID-19. Our finding of accessibility of telehealth for those with comorbidities highlights in part medical decision-making from both providers and patients to prevent and reduce the risk of COVID-19 among those most at risk. Medicare recently reported that among Part B beneficiaries with COVID-19, more than 50% have hypertension, hyperlipidemia, or diabetes (3), which further affirms the need for telehealth services in this population during the pandemic. These findings appear to show that those at most risk of adverse health outcomes from COVID-19 have transitioned to telehealth during the COVID-19 pandemic; however, the prevalence of availability and use of telehealth that we report here should be investigated further to ensure that distribution of its accessibility and use are matching the needs for various at-risk groups.

We found a significant association between beneficiaries who had participated previously in video, voice, or conference calls over the internet and those who reported that their usual providers offered telehealth to replace a regularly scheduled appointment. This finding is not surprising, considering that providers are probably more willing to offer telehealth services to older adults who are already comfortable using technology. Therefore, patient education and training related to telehealth is needed (ie, installing software and apps, or launching web conferencing platforms) (28). Additionally, health care systems and providers need to be adequately equipped, trained to be telehealth-ready, and encouraged to incorporate telehealth as part of routine care (27,28).

With the increased use of telehealth, many questions remain that warrant a serious discussion (eg, whether the policy changes on telehealth made during the current health crisis should be made permanent, the quality and privacy of using telehealth). Decision makers and others (ie, payers, providers, patients, and health care systems) should consider all these elements, especially if the goal is to have the use of telehealth services extend beyond the COVID-19 pandemic.

### Limitations

Our study has several limitations. We included only community-dwelling Medicare beneficiaries aged 65 years or older; therefore, our findings may not be generalizable to people who live in long-term care facilities and to non-Medicare populations. Additionally, this is a cross-sectional study, so the results represent associations rather than causation. With the use of a self-reported survey, estimates are subject to recollection errors and biases. Ideally, we would have preferred to use claims data, but they were not available from the MCBS COVID-19 Summer Supplemental Public Use File. Nevertheless, our estimates in terms of accessibility of telehealth are consistent with studies related to COVID-19 (1,12,13). Limited covariates were available; variables such as education attainment, disability status, employment information, and attitudes toward health care were not available, which could have affected our findings. Additionally, the clinical practice’s characteristics (eg, solo vs group practice, size of the practice, location, setting) were not available, and these characteristics would also probably affect the availability and accessibility of telehealth services among beneficiaries. For example, information related to clinician specialties is an important factor, because some medical care requires in-person examinations, whereas other health care services are more easily conducted by using telehealth. With older adults and racial and ethnic minority populations disproportionately affected by COVID-19, further research that focuses on interaction effects between digital access and literacy and demographic or subgroup analysis by race/ethnicity and age group are warranted to better understand their relationships with accessibility of telehealth.

## Conclusion

We found that, in general, Medicare beneficiaries most at risk of COVID-19 exposure were associated with having their usual providers offer telehealth to replace a regularly scheduled appointment. This is probably due in part to approval of telehealth policy changes, increased awareness of availability of telehealth, willingness to adopt telehealth, and advocacy of the need for telehealth to reduce the risk of COVID-19. However, sociodemographic disparities (ie, sex, residing area, income level, census region) in accessibility of telehealth services remain a concern, especially considering the obvious benefits of telehealth to increasing access to care. More outreach efforts are needed for those in at-risk groups, which can potentially lead to telehealth continuing as a method of care delivery, even after the COVID-19 pandemic.
